# Concomitant inpatient prescribing of strong opioids with sedatives: Associations with comorbid conditions

**DOI:** 10.1002/prp2.717

**Published:** 2021-02-07

**Authors:** Ray J. Li, Gillian E. Caughey, Sepehr Shakib

**Affiliations:** ^1^ Department of Clinical Pharmacology Royal Adelaide Hospital Adelaide Australia; ^2^ Discipline of Pharmacology Adelaide Medical School The University of Adelaide Adelaide Australia; ^3^ Registry of Senior Australians South Australian Health and Medical Research Institute Adelaide Australia; ^4^ Division of Health Sciences University of South Australia Adelaide Australia

**Keywords:** hypnotics and sedatives, inpatients, opioid, pain management, prescriptions, respiration

## Abstract

Co‐prescribing of opioids and sedatives is a known risk factor for opioid‐induced ventilatory impairment (OIVI). Prevalence data for sedative and opioid co‐prescription in inpatients in Australia are unknown. Our objective was to determine the prevalence of inpatient sedative and opioid co‐prescribing and to identify factors associated with co‐prescription. We conducted a retrospective cross‐sectional study from July 2017 to October 2017 across four South Australian hospitals utilizing a centralized electronic health record. Multivariate analysis was used to identify characteristics predictive of co‐prescribing of a strong opioid (fentanyl, hydromorphone, morphine, and oxycodone) and sedative medications (benzodiazepines, antiepileptics, antipsychotics, and tricyclic antidepressants). Of the 6170 inpatients, 2795 (45.3%) were prescribed a strong opioid and of those, 1889 (30.6% of all inpatients) were co‐prescribed a sedative. Of those prescribed a strong opioid, five (0.18%) developed OIVI. Patients prescribed a strong opioid had a 27–77% increased likelihood of being prescribed a sedative. Factors predictive of sedative co‐prescribing included the presence of disease of the central nervous system adjusted OR (aOR) 8.66 [95% CI 5.83–12.9] and respiratory disease aOR 1.42 [95% CI 1.17–1.72]. Nearly, one third of all hospital inpatients were co‐prescribed a strong opioid and a sedative medication. Patients with comorbidities resulting in increased risk of respiratory depression/OIVI were more likely to have sedative co‐prescription. Clinicians should be aware of the effects of high‐risk medications and ensure that systems and monitoring are in place that help mitigate adverse outcomes.

**Abbreviations**
aOR: adjusted OR
DBI: drug burden index
EHR: electronic health record
OIVI: opioid‐induced ventilatory impairment

## 1 INTRODUCTION

Opioid prescribing in the community has increased 15‐fold from 500 000 opioid dispensing episodes in 1992 to 7.5 million in 2012 in Australia and is now one of the leading causes of prescription overdose and death worldwide.^1^, ^2^ While Australian studies have previously investigated the prevalence of opioid use in the community,^3^ no studies thus far have investigated the rates of inpatient opioid prescription in Australia. The use of sedating medicines, mainly benzodiazepines, is commonly used concomitantly in patients who receive opioids, and benzodiazepines are implicated in 30.1% of all opioid‐related overdose deaths.^4^, ^5^, ^6^ Opioid‐induced ventilatory impairment (OIVI) from suppression of the respiratory centers in the brain is a potentially fatal adverse effect of opioid use.^7^, ^8^ Sedative medications potentiate these central nervous system depression effects, resulting in increased risk of respiratory depression and death.^9^, ^10^

There are a number of medications with sedative properties, including psychotropic medications, and in Australia their use has increased 58% from 82.4 defined daily doses/1000 population/day (DDDs/1000/day) in 2000 to 130.4 DDDs/1000/day in 2011.^11^ A more recent study investigating individual‐level dispensing claims in Australia showed a 2.6% relative increase in the annual prevalence of psychotropic medication use between 2007 and 2015.^12^ Thus far, no studies have investigated the prevalence of inpatient psychotropic use in Australia. Along with being used to treat psychiatric conditions, psychotropics are occasionally used as adjuvant analgesics in the treatment of chronic pain.^13^, ^14^ Concomitant prescription of opioids and psychotropic medications, such as benzodiazepines, is not uncommon in certain populations such as veterans,^15^ and an Australian population‐based study of over 600 000 people who use opioids showed that, while declining, 12.7% were concomitantly dispensed a benzodiazepine in 2012.^16^ Concurrent prescription of opioids, psychotropics, and other medications with sedative properties increases the drug burden index (DBI).^17^ While higher inpatient DBI has not been associated with a higher mortality,^18^ it has been associated with higher risk of falls,^18^ decreased physical functioning, and increased length of stay.^19^

Opioids are the mainstay of analgesia for the treatment of acute postoperative pain.^20^ Strong opioids, defined as those used to treat moderate to severe pain, such as morphine, oxycodone, and fentanyl, are more likely to be associated with increased risk of adverse outcomes.^21^ There is a paucity of prevalence data on strong opioid and sedative co‐prescription. The only inpatient study thus far has demonstrated that 16.9% of all medical and 32.6% of all surgical patients were co‐prescribed a sedative in a multicenter United States study.^22^ Current prevalence data for inpatient sedative medications and opioid co‐prescribing in Australia are unknown. Therefore, to identify prescribing patterns that facilitate potential increased risk of OIVI, the aim of this study was to investigate inpatient co‐prescription of sedative medications with strong opioids in hospitalized patients and to identify the demographic and clinical factors potentially associated with this prescribing.

## 2 METHODS

### 2.1 Data source and cohort selection

This study was approved by the South Australian Department for Health and Ageing Human Research Ethics Committee.

This study used inpatient data from the Allscripts Sunrise electronic health record (EHR) (Allscripts) from four public hospitals in South Australia. The hospitals included the Queen Elizabeth Hospital, a large metropolitan adult general hospital, the Port Augusta Hospital, a small general country hospital, Noarlunga Hospital, a small metropolitan community general hospital, and Repatriation General Hospital, a metropolitan hospital specializing in management of older patients. Data on demographics, admission unit, documented comorbidities, and all medications prescribed during inpatient stay were collected for this study.

A retrospective cross‐sectional study of all inpatients admitted to four public hospitals within South Australia over a 4‐month period from July 1, 2017 to October 31, 2017 was conducted. Patients were excluded if they were aged less than 18 years, if prescribed methadone or oral buprenorphine, which indicated opioid substitution therapy, or if they were only managed in the emergency department.

### 2.2 Measures and definitions

Medications were classified according to the Anatomical Therapeutic Chemical (ATC) classification system by the World Health Organization.^23^ Opioid medications were defined as opioids under Class N (narcotics and other sedatives) of high‐risk medications, as defined by the South Australian Department for Health and Ageing Safety and Quality Unit (Table S1). This document was chosen as it is the local guideline that states to avoid using sedatives with opioids and defines which medications to avoid. We also included tapentadol, which is used in the South Australian health system. Our assessment of opioids only included strong opioids, as they confer a higher risk for opioid‐related adverse effects (Table S1). Strong opioids included fentanyl, hydromorphone, morphine, and oxycodone. Strong opioids were further classified as immediate or slow release formulations.

Sedative medications were defined as the non‐opioid medications under Class N of high‐risk medications, and psychotropic medications (Table S1). Sedatives were defined as selected medications in ATC classes N03 (antiepileptics), N05 (psycholeptics), and N06 (psychoanaleptics). The included medications were categorized into benzodiazepines, antiepileptics, antipsychotics, and tricyclic antidepressants. Benzodiazepines were further broken down into short‐acting (duration of action <12 h), medium‐acting (duration of action 12–24 h), and long‐acting (duration of action >24 h). Medications were considered concomitant if they were prescribed both medications at any point during their hospital admission, as our previous work had shown that the vast majority of opioids were prescribed as required (PRN), hence were available to be administered, throughout the admission.^24^ As medications prescribed intraoperatively and in the postoperative recovery setting are not coded into our EHR dataset, medications included in our study are limited to those prescribed on the wards. We excluded opioid and sedative medications that were prescribed as an infusion, as these are used exclusively in a palliative care and critical care setting. We included patients on the palliative care ward in our analysis, as outlying patients not under the care of the palliative care team are often situated on the ward.

In the hospitals included in this study, presence of OIVI is monitored by regular sedation scores: 0 = awake, alert when approached; 1 = easy to rouse; 2 = easy to rouse, difficulty staying awake; 3 = difficult to rouse. A sedation score greater than or equal to two triggers an alert for medical review, and naloxone is only administered in the setting of a resuscitation attempt. One of the authors further investigated these naloxone events and only included those where sedation and respiratory depression were reversed within minutes of naloxone administration, indicating the presence of OIVI.

Comorbidities were defined from both what was documented in the problem list of the EHR and using the Rx‐Risk Comorbidity Index, a validated medication‐based comorbidity index designed to map medicines to corresponding medical conditions at the ATC classification level.^25^ Central nervous system disease was defined as having any of the following documented in the problem list: stroke, epileptic seizure, and epilepsy. Dementia was defined as Alzheimer's disease, vascular dementia, or dementia from other medical conditions. Respiratory disease was defined as having any of obstructive sleep apnea, chronic obstructive pulmonary disease, asthma, restrictive lung disease, or pneumonia. Risk of falls was defined as having a documented risk of falls, previous fall, or falls at presentation. Affective psychiatric illness was defined as having any of adjustment disorder, depression, anxiety disorder, bipolar disorder, post‐traumatic stress disorder, functional disorder, social phobia, suicide risk, intentional self‐harm or neglect, mania, obsessive compulsive disorder, agoraphobia, panic disorder, conversion disorder, or schizoaffective disorder. Psychotic illness was defined as any of psychotic disorder, psychosis, schizoaffective disorder, schizophrenia, or delusional disorder.

### 2.3 Statistical analysis

Univariate analysis was conducted to examine patient demographics and co‐prescription of a sedative with a strong opioid using Chi‐square tests for statistical significance. Odds ratios (OR) and 95% confidence intervals (CI) were calculated for co‐prescription of a sedative drug in patients who were prescribed a strong opioid. Multivariate logistic regression analyses were used to determine demographic and clinical characteristics associated with being prescribed a sedative in patients who were prescribed a strong opioid. Backward stepwise multivariate logistic regression was used with all variables with a *p*‐value <.20 in univariate analysis included and only those where *p* < .05 were included in the final model. Data were analyzed using SPSS version 25 (IBM Corp).

## 3 RESULTS

A total of 6170 inpatients who met the study inclusion criteria were identified over the 4‐month study period. The median age of the cohort was 70 (IQR 53–83) years old and 51.1% were male. In the study cohort, 2795 (45.3%) patients were prescribed a strong opioid, and of those 1889 (30.6% of all inpatients) were prescribed both an opioid and a sedative. Median length of stay was 3.2 (IQR 1.0–8.1) days for patients not prescribed a strong opioid, 4.5 (IQR 1.8–10.8) days for patients prescribed a strong opioid, and 5.5 (IQR 2.1–11.9) for patients prescribed both a strong opioid and a sedative. Three hundred and three (9.0%) patients not prescribed a strong opioid, 462 (16.5%) patients prescribed a strong opioid, and 360 (19.1%) patients prescribed both a strong opioid and a sedative were additionally prescribed a weak opioid such as codeine. Of those prescribed a strong opioid, 2600 (93.0%) were prescribed PRN. Documentation of patient comorbidities was not documented for 2127 (34.5%) patients, and for these, comorbidities were derived from their prescribed medications using Rx‐Risk Comorbidity Index, a validated pharmacy‐based measure of comorbidity that maps medicines to medical conditions.^25^ Comorbidities which were statistically significant in the univariate analysis were central nervous system disease, renal disease, dementia, congestive heart failure, respiratory disease, chronic obstructive pulmonary disease, obstructive sleep apnea, any malignancy, risk of falls, affective disorders, and psychotic disorders (Table 1). Of all patients prescribed a strong opioid, five (0.18%) were administered naloxone and had an immediate positive response, which suggests the development of OIVI. All five were co‐prescribed both a strong opioid and a sedative medication.

**TABLE 1:** Patient demographics and comorbid conditions, n (%).

	Overall, n = 6170	No strong opioid n = 3375 (54.7%)	Strong opioid n = 2795 (45.3%)	Strong opioid and sedative n = 1889 (30.6%)
Age, years, median (IQR)	70 (53–83)	70 (53–83)	70 (53–83)	70 (54–82)
Gender (% male)	3155 (51.1)	1768 (52.4)	1387 (49.6)	918 (48.6)
Length of stay, days, median (IQR)	3.9 (1.4–9.3)	3.2 (1.0–8.1)	4.5 (1.8–10.8)	5.5 (2.1–11.9)
Service				
Medicine	4171 (67.6)	2595 (76.9)	1576 (56.4)	1036 (54.8)
Surgery	1256 (20.4)	233 (6.9)	1023 (36.6)	661 (35.0)
Mental health	595 (9.6)	539 (16.0)	56 (2.0)	56 (3.0)
Palliative care	148 (2.4)	8 (0.2)	140 (5.0)	136
Weak opioid	765 (12.4)	303 (9.0)	462 (16.5)	360 (19.1)
Comorbid conditions^a^, ^b^				
Central nervous system disease^a^	1065 (17.3)	561 (16.6)	504 (18.0)	476 (44.7)
Renal disease^a^	315 (5.1)	182 (5.4)	133 (4.8)	105 (33.3)
Dementia^a^	223 (3.6)	140 (4.1)	83 (3.0)	69 (30.9)
Diabetes mellitus^a^	1481 (24.0)	865 (25.6)	616 (22.0)	453 (30.6)
Congestive heart failure^a^	1549 (25.1)	780 (23.1)	769 (27.5)	573 (37.0)
Respiratory disease^a^	2010 (32.6)	1126 (33.4)	884 (31.6)	654 (32.5)
Chronic obstructive pulmonary disease^b^	407 (10.1)	251 (11.2)	156 (8.7)	122 (30.0)
Obstructive sleep apnea^b^	208 (5.1)	138 (6.1)	70 (3.9)	56 (26.9)
Liver cirrhosis^b^	46 (1.1)	19 (0.8)	27 (1.5)	18 (39.1)
Myocardial infarction^b^	158 (3.9)	94 (4.2)	64 (3.6)	45 (28.5)
Cerebrovascular disease^b^	75 (1.9)	43 (1.9)	32 (1.8)	28 (37.3)
Any malignancy^b^	350 (8.7)	147 (6.6)	203 (11.3)	149 (42.6)
Risk of falls^b^	185 (4.6)	82 (3.7)	103 (5.7)	73 (39.5)
Affective disorders^b^	688 (17.0)	466 (20.8)	222 (12.3)	200 (29.1)
Psychotic disorders^b^	216 (5.3)	187 (8.3)	29 (1.6)	26 (12.0)

^a^ Comorbidity was defined from recorded conditions within the medical record and medications using the Rx‐Risk Comorbidity Index, n = 6170.

^b^ Comorbidity defined from recorded conditions within the medical record, n = 4043.

Patients prescribed a strong opioid were 44% more likely (OR 1.44 [95% CI 1.24–1.68]) to be co‐prescribed a long‐acting benzodiazepine than patients not prescribed a strong opioid. Patients prescribed a strong opioid were more than twice as likely (OR 2.16 [95% CI 1.90–2.45]) to be co‐prescribed a short‐acting benzodiazepine, and less likely to be co‐prescribed a medium‐acting benzodiazepine (OR 0.18 [95% CI 0.14–0.24]). Prescribing of a strong opioid was also associated with a 46–77% increased likelihood of being co‐prescribed an antiepileptic, antipsychotic, or tricyclic antidepressant (Table 2). Of the 972 patients co‐prescribed a benzodiazepine, the majority (65.2%, n = 729) were co‐prescribed one benzodiazepine, 26.1% were prescribed two benzodiazepines (n = 233), and 8.3% (n = 10) were prescribed three. Of those prescribed a strong opioid, patients prescribed a long‐acting benzodiazepine had an 84% increased likelihood of being prescribed a slow‐release opioid formulation as opposed to immediate release (OR 1.84 [95% CI 1.43–2.35]).

**TABLE 2:** Proportion and type of sedative co‐prescribing in patients prescribed a strong opioid (n = 2795).

	Type of sedative co‐prescribed in patients prescribed a strong opioid n = 2795	Unadjusted odds ratio (95% Confidence Interval) of co‐prescription of the sedative class in those prescribed opioids	*p*‐value
Benzodiazepine^a^	972 (34.8)	1.27 (1.14–1.41)	<.001
Short‐acting benzodiazepine	766 (27.4)	2.16 (1.90–2.45)	<.001
Medium‐acting benzodiazepine	61 (2.2)	0.18 (0.14–0.24)	<.001
Long‐acting benzodiazepine	398 (14.2)	1.44 (1.24–1.68)	<.001
Antiepileptic	505 (18.1)	1.46 (1.27–1.68)	<.001
Antipsychotic	930 (33.3)	1.74 (1.55–1.94)	<.001
Tricyclic antidepressant	138 (4.9)	1.77 (1.36–2.31)	<.001

^a^ Benzodiazepine subclasses sum to greater than overall benzodiazepines, as patients are concomitantly prescribed short‐acting, medium‐acting, and long‐acting benzodiazepines.

Table 3 shows demographic and comorbid conditions that were independently associated with sedative co‐prescription in patients prescribed a strong opioid. Multivariate analysis showed admission to a surgical (adjusted odds ratio (aOR) 1.23 [95% CI: 1.03–1.46]) or palliative care ward (aOR: 11.5 [95% CI: 4.17–31.6]), presence of CNS disease (aOR 8.66 [95% CI: 5.83–12.9]), and respiratory disease (aOR 1.42 [95% CI: 1.17–1.72]) were independently associated with a greater risk of being co‐prescribed a sedative in patients who were prescribed a strong opioid (Table 3).

**TABLE 3:** Independent predictors of being co‐prescribed a sedative in those prescribed a strong opioid (n = 2795) by patient demographics and comorbid conditions^a^.

Patient characteristic	Odds ratio (95% confidence interval)	*p*‐value
Ward		
Medicine	1.00	
Surgery	1.23 (1.03–1.46)	.023
Palliative care	11.5 (4.17–31.6)	<.001
CNS disease		
No CNS disease	1.00	
CNS disease	8.66 (5.83–12.9)	<.001
Respiratory disease		
No respiratory disease	1.00	
Respiratory disease	1.42 (1.17–1.72)	<.001

Model Chi‐square =339.1; model Chi‐square *p*‐value <.001; c‐statistic =0.663. CNS, Central nervous system.

^a^ Backward stepwise multivariate logistic regression: all variables with a *p*‐value <.20 in univariate analysis were included; only those where *p* < .05 were included in the final model.

## 4 DISCUSSION

Our findings show that nearly one third (30.6%) of all inpatients admitted to South Australian public hospitals over a 4‐month period were co‐prescribed both a strong opioid and a sedative medication. These findings are concordant with a recent United States study of 21 276 691 patients that reported 16.9% of medical patients and 32.6% of surgical patients were prescribed both an opioid and a sedative.^22^ In our study, patients prescribed a strong opioid had a 27%–77% increased likelihood of being prescribed a sedative. Patients prescribed a strong opioid also had a decreased likelihood of being prescribed a medium‐acting benzodiazepine. This is likely due to the inclusion of lorazepam whose use in the South Australian Medicines Formulary, a list of medicines approved for use in South Australian public hospitals, is limited to specific indications, such as catatonia and certain types of epilepsy. It is possible that this decreased likelihood is due to there being little overlap between these conditions and the presence of acute pain requiring opioids. However, further research is required to elaborate on this finding.

An area of concern is the co‐prescription of opioids and sedatives in those with comorbidities that increase the risk of OIVI. Previous studies have identified conditions such as respiratory disease, neurological disorders, and renal disease as risk factors for OIVI.^26^, ^27^ Our study found that in patients prescribed a strong opioid, sedative co‐prescription was more likely in patients who had CNS or respiratory disease. Previous studies have found associations between concurrent benzodiazepine and higher dose opioid prescription with increased mortality in patients with severe respiratory disease.^28^ Further dose‐level analysis suggested that it was likely a result of the medication co‐prescription as opposed to a reflection of a more severe underlying illness.^28^ A study reviewing over 4000 patients found that the most frequent cause of all adverse drug events was analgesics, with opioids accounting for at least 18% of these events.^29^ Sedatives were the third most frequent cause, with almost a third of all adverse drug events being preventable. Clinicians should therefore balance the benefits of symptomatic relief against the increased risk of adverse drug events.

Our findings that the patients who are at higher risk of OIVI are also at risk of sedative and opioid co‐prescribing indicate the need for increased prescriber awareness and consideration. Further, 19.1% of those who were concurrently prescribed a strong opioid and sedative medication were also on a weak opioid. This highlights the significantly high overall utilization of opioid medications while patients are admitted during hospitalization placing patients at potential increased risk of opioid‐related adverse effects. If treatment with concurrent sedative and opioid medications is necessary, considerations for safer practice such as beginning treatment with lower dose opioids^28^ is indicated. The indications for a sedative and opioid co‐prescription may be connected. Indeed, fatigue and sleep issues as somatic comorbidities of pain have been previously described.^30^ Clinicians may view that the benefits of symptom relief, particularly in the elderly, from sedative co‐prescription outweigh the risks and decide that it is appropriate. The indication for a sedative medication, such as a mental health disorder, may also be independent from the indication for opioids, such as acute pain. In many of these instances, there may not be discretion in the choice of co‐prescribing of opioids and sedatives, for example, in the use of adjuvant therapies for chronic pain such as gabapentinoids, or patients with long‐standing use of sedatives such as benzodiazepines or antipsychotics who present to hospital in acute pain. The reduction or cessation of these sedatives may be associated with more harm in such patients, and their acute pain may warrant management with strong opioids. In such cases, other practices including regular sedation score and vital sign monitoring, and naloxone standing orders for nursing staff are prudent for safe opioid use.

A United States study^22^ found that in both medical and surgical patients, the concurrent use of opioids and sedatives conveyed the highest risk of developing cardiopulmonary and respiratory arrest. In our cohort, five inpatients (0.18% of all patients prescribed a strong opioid) developed OIVI and all were co‐prescribed a sedative medication. This is consistent with previous studies showing OIVI incidence of between 0.09% and 0.2%.^31^, ^32^ The potential for OIVI and subsequent cardiorespiratory arrest to be fatal indicate the need for greater care in prescribing and to ensure adequate monitoring when administering both an opioid and a sedative. We found an increased length of stay for patients who were prescribed both compared to those who were not. This is in line with previous studies,^33^ which have also demonstrated increased cost associated with opioid adverse drug effects, even when it is non‐lethal. Conversely, it may also be due to patients with greater length of stay having greater opportunity to be prescribed both medications, or may represent the increased complexity of these patients.

This study has several limitations. Documentation of patient comorbidities was not documented for 2127 (34.5%) patients. Mapping the Rx‐Risk Comorbidity Index to the ATC codes partially remedied the missing information due to medication information being available for all patients. Data, such as potentially relevant medications administered intraoperatively or in recovery in surgical patients, were not available in the Allscripts EHR. Our study did not consider medication dosage due to the wide variability in opioid dose response between patients.^34^ Our study's definition of concomitant medications was also unable to distinguish whether the patient was administered concomitant medications at the same time or whether they were administered at different points during their stay. It was also unable to distinguish medications that were discontinued partway during their stay and may not have had a duration of overlap in prescription. While some sedative drugs, such as benzodiazepines, may be less sedative than others, such as antipsychotics, the differences in individual sedative effects between these drugs are difficult to define. Therefore, to investigate the potential for increased risk of OIVI, all were included. The indication for prescription was not available in our dataset, hence our study was unable to evaluate the appropriateness of sedative and opioid co‐prescription. As the patients in our study population were admitted over a 4‐month period between July 2017 and October 2017, they may not represent the admission characteristics of patients admitted over the rest of the year.

In conclusion, 30.6% of all inpatients were co‐prescribed a sedative and strong opioid. Patients at increased risk of opioid‐related harms, such as patients with CNS disease and respiratory disease, were actually more likely to have a concomitant prescription of sedatives in those prescribed a strong opioid. Clinicians should be aware of the effects of high‐risk medications and ensure that systems and monitoring are in place that help mitigate adverse outcomes. Further study in this field includes investigating adverse outcomes related to sedative and opioid co‐prescription, looking at the trends in inpatient prescriptions over time and more focused analysis in subgroups.

## DISCLOSURE

RJ Li, GE Caughey, and S Shakib declare that they have no financial, personal, or potential conflict of interest that might be relevant to the contents of this manuscript.

## AUTHOR CONTRIBUTIONS

RJL contributed to the study design and analysis plan, data and statistical analysis, wrote the manuscript, and reviewed/edited the manuscript. GEC contributed to the study design and analysis plan, and reviewed/edited the manuscript. SS contributed to the study design and analysis plan, and reviewed/edited the manuscript.

## ETHICS APPROVAL

This study was approved by the South Australian Department for Health and Ageing Human Research Ethics Committee.

## Supporting information

Table S1–S2

## Data Availability

The data that support the findings of this study are available from the corresponding author upon reasonable request.
