# Supplementary material for: Concomitant inpatient prescribing of strong opioids with sedatives: Associations with comorbid conditions
Source: Pharmacol Res Perspect. 2021 Feb 7;9(1):e00717. doi: 10.1002/prp2.717 (PMC7868063; doi:10.1002/prp2.717)
Supplement: Supplementary file 1 — Table S1–S2 [file PRP2-9-e00717-s001.docx]

**Title:** Concomitant Inpatient Prescribing of Strong Opioids with Sedatives: Associations with Comorbid Conditions

**Running Title:** Concomitant inpatient prescribing of opioids and sedatives

**Authors:** Ray J. Li^1,2^, Gillian E. Caughey^1-4^, Sepehr Shakib^1,2^

**Affiliations:**

^1^Department of Clinical Pharmacology, Royal Adelaide Hospital, Adelaide, Australia, 5000

^2^Discipline of Pharmacology, Adelaide Medical School, The University of Adelaide, Adelaide, Australia, 5000

^3^Registry of Senior Australians, South Australian Health and Medical Research Institute, Adelaide, Australia, 5000

^4^Division of Health Sciences, University of South Australia, Adelaide, Australia, 5000

**Corresponding Author:**

Ray J. Li

Clinical Pharmacology, Royal Adelaide Hospital

**E-mail:** ray.li@student.adelaide.edu.au

**Journal Name:** Pharmacology Research & Perspectives  **Number of supplementary tables:** 2

**SUPPLEMENTARY APPENDIX**

**Supplementary Table 1: Medications list**

| **Opioids** |  |
| --- | --- |
| Weak opioids | Buprenorphine, codeine, tramadol, tapentadol |
| Strong opioids | Fentanyl, fentanyl citrate, hydromorphone, morphine, oxycodone |
| **Sedatives** |  |
| Short-acting benzodiazepines (duration of action < 12 hours) under class N of high risk medications | Alprazolam, oxazepam, temazepam, midazolam, zolpidem, zopiclone |
| Medium-acting benzodiazepines (duration of action 12-24 hours) under class N of high risk medications | Bromazepam, lorazepam |
| Long-acting benzodiazepines (duration of action > 24 hours) under class N of high risk medications | Clobazam, clonazepam, diazepam, flunitrazepam, nitrazepam |
| Antiepileptics (N03) | Phenobarbital, primidone, phenytoin, ethosuximide, clonazepam, carbamazepine, oxcarbazepine, tiagabine, valproate, vigabatrin, gabapentin, lacosamide, lamotrigine, levetiracetam, perampanel, pregabalin, sulthiame, topiramate, zonisamide |
| Psycholeptics (N05) | Amisulpride, aripiprazole, asenapine, clozapine, lurasidone, olanzapine, paliperidone, quetiapine, risperidone, ziprasidone, chlorpromazine, flupenthixol decanoate, haloperidol, pericyazine, trifluoperazine, zuclopenthixol, lithium carbonate, droperidol, brexpiprazole, buspirone |
| Psychoanaleptics (N06) | Moclobemide, phenelzine, tranylcypromine, mianserin, mirtazapine, desvenlafaxine, duloxetine, reboxetine, venlafaxine, citalopram, escitalopram, fluoxetine, fluvoxamine, paroxetine, sertraline, amitriptyline, clomipramine, dosulepin, doxepin, imipramine, nortriptyline |

**Supplementary Table 2: Predictors of sedative co-prescription in patients prescribed a strong opioid by sedative class**

|  | | **Overall (% prescribed the sedative class in those already prescribed a strong opioid)** | **Odds ratio of being co-prescribed the sedative class (95% Confidence Interval)** | **p-value** |
| --- | --- | --- | --- | --- |
| **Short acting benzodiazepine (n=766)** | Age <75 years | 347 (21.5) | 1.00 |  |
|  | Age ≥75 years | 419 (35.5) | 2.01 (1.70 – 2.38) | < 0.001 |
|  | Female | 398 (28.3) | 1.00 |  |
|  | Male | 368 (26.5) | 0.92 (0.78 – 1.08) | 0.304 |
|  | Surgery | 125 (12.2) | 1.00 |  |
|  | Medicine | 497 (31.5) | 3.31 (2.67 – 4.10) | < 0.001 |
|  | Mental Health | 33 (58.9) | 10.3 (5.86 – 18.1) | < 0.001 |
|  | Palliative care | 111 (79.3) | 27.5 (17.5 – 43.1) | < 0.001 |
|  | No CNS disease^†^ | 519 (22.7) | 1.00 |  |
|  | CNS disease | 247 (49.0) | 3.28 (2.69 – 4.01) | < 0.001 |
|  | No respiratory disease^†^ | 432 (22.6) | 1.00 |  |
|  | Respiratory disease | 334 (37.8) | 2.08 (1.75 – 2.47) | < 0.001 |
|  | No renal disease^†^ | 710 (26.7) | 1.00 |  |
|  | Renal disease | 56 (42.1) | 2.00 (1.40 – 2.85) | < 0.001 |
|  | No dementia^†^ | 718 (26.5) | 1.00 |  |
|  | Dementia | 48 (57.8) | 3.81 (2.44 – 5.94) | < 0.001 |
|  | No diabetes mellitus^†^ | 564 (25.9) | 1.00 |  |
|  | Diabetes mellitus | 202 (32.8) | 1.40 (1.15 – 1.70) | 0.001 |
|  | No OSA^‡^ | 511 (29.6) | 1.00 |  |
|  | OSA | 22 (31.4) | 1.09 (0.65 – 1.83) | 0.736 |
|  | No liver cirrhosis^‡^ | 519 (29.3) | 1.00 |  |
|  | Liver cirrhosis | 14 (51.9) | 2.60 (1.21 – 5.57) | 0.011 |
|  | No risk of falls^‡^ | 503 (29.7) | 1.00 |  |
|  | Risk of falls | 30 (29.1) | 0.98 (0.63 – 1.51) | 0.909 |
| **Medium acting benzodiazepine (n=61)** | Age <75 years | 45 (2.8) | 1.00 |  |
|  | Age ≥75 years | 16 (1.4) | 0.48 (0.27 – 0.85) | 0.010 |
|  | Female | 30 (2.1) | 1.00 |  |
|  | Male | 31 (2.2) | 1.05 (0.63 – 1.74) | 0.850 |
|  | Surgery | 5 (0.5) | 1.00 |  |
|  | Medicine | 37 (2.3) | 4.90 (1.92 – 12.5) | < 0.001 |
|  | Mental Health | 18 (32.1) | 96.4 (34.0 – 273.5) | < 0.001 |
|  | Palliative care | 1 (0.7) | 1.47 (0.17 – 12.6) | 0.727 |
|  | No CNS disease^†^ | 42 (1.8) | 1.00 |  |
|  | CNS disease | 19 (3.8) | 2.10 (1.21 – 3.64) | 0.007 |
|  | No respiratory disease^†^ | 33 (1.7) | 1.00 |  |
|  | Respiratory disease | 28 (3.2) | 1.86 (1.12 – 3.10) | 0.015 |
|  | No renal disease^†^ | 59 (2.2) | 1.00 |  |
|  | Renal disease | 2 (1.5) | 0.67 (0.16 – 2.79) | 0.583 |
|  | No dementia^†^ | 59 (2.2) | 1.00 |  |
|  | Dementia | 2 (2.4) | 1.11 (0.27 – 4.62) | 0.886 |
|  | No diabetes mellitus^†^ | 46 (2.1) | 1.00 |  |
|  | Diabetes mellitus | 15 (2.4) | 1.16 (0.64 – 2.09) | 0.627 |
|  | No OSA^‡^ | 46 (2.7) | 1.00 |  |
|  | OSA | 1 (1.4) | 0.53 (0.07 – 3.90) | 0.526 |
|  | No liver cirrhosis^‡^ | 47 (2.7) | 1.00 |  |
|  | Liver cirrhosis | 0 (0) | - | - |
|  | No risk of falls^‡^ | 44 (2.6) | 1.00 |  |
|  | Risk of falls | 3 (2.9) | 1.13 (0.34 – 3.69) | 0.844 |
| **Long acting benzodiazepine (n=398)** | Age <75 years | 203 (12.6) | 1.00 |  |
|  | Age ≥75 years | 195 (16.5) | 1.38 (1.11 – 1.70) | 0.003 |
|  | Female | 187 (13.3) | 1.00 |  |
|  | Male | 211 (15.2) | 1.17 (0.95 – 1.45) | 0.144 |
|  | Surgery | 79 (7.7) | 1.00 |  |
|  | Medicine | 213 (13.5) | 1.87 (1.42 – 2.45) | < 0.001 |
|  | Mental Health | 27 (48.2) | 11.1 (6.28 – 19.7) | < 0.001 |
|  | Palliative care | 79 (56.4) | 15.5 (10.3 – 23.2) | < 0.001 |
|  | No CNS disease^†^ | 151 (6.6) | 1.00 |  |
|  | CNS disease | 247 (49.0) | 13.6 (10.7 – 17.3) | < 0.001 |
|  | No respiratory disease^†^ | 222 (11.6) | 1.00 |  |
|  | Respiratory disease | 176 (19.9) | 1.89 (1.52 – 2.35) | < 0.001 |
|  | No renal disease^†^ | 374 (14.0) | 1.00 |  |
|  | Renal disease | 24 (18.0) | 1.35 (0.85 – 2.12) | 0.198 |
|  | No dementia^†^ | 372 (13.7) | 1.00 |  |
|  | Dementia | 26 (31.3) | 2.87 (1.78 – 4.62) | < 0.001 |
|  | No diabetes mellitus^†^ | 309 (14.2) | 1.00 |  |
|  | Diabetes mellitus | 89 (14.4) | 1.02 (0.79 – 1.32) | 0.867 |
|  | No OSA^‡^ | 268 (15.1) | 1.00 |  |
|  | OSA | 10 (14.3) | 0.93 (0.47 – 1.84) | 0.832 |
|  | No liver cirrhosis^‡^ | 268 (15.1) | 1.00 |  |
|  | Liver cirrhosis | 5 (18.5) | 1.28 (0.48 – 3.40) | 0.626 |
|  | No risk of falls^‡^ | 250 (14.7) | 1.00 |  |
|  | Risk of falls | 23 (22.3) | 1.66 (1.03 – 2.70) | 0.037 |
| **Antiepileptic (n=505)** | Age <75 years | 284 (17.6) | 1.00 |  |
|  | Age ≥75 years | 221 (18.7) | 1.08 (0.89 – 1.31) | 0.448 |
|  | Female | 268 (19.0) | 1.00 |  |
|  | Male | 237 (17.1) | 0.88 (0.72 – 1.06) | 0.181 |
|  | Surgery | 84 (8.2) | 1.00 |  |
|  | Medicine | 351 (22.3) | 3.20 (2.49 – 4.12) | < 0.001 |
|  | Mental Health | 30 (53.6) | 12.9 (7.29 – 22.8) | < 0.001 |
|  | Palliative care | 40 (28.6) | 4.47 (2.91 – 6.87) | < 0.001 |
|  | No CNS disease^†^ | 251 (11.0) | 1.00 |  |
|  | CNS disease | 254 (50.4) | 8.26 (6.64 – 10.3) | < 0.001 |
|  | No respiratory disease^†^ | 309 (16.2) | 1.00 |  |
|  | Respiratory disease | 196 (22.2) | 1.48 (1.21 – 1.80) | < 0.001 |
|  | No renal disease^†^ | 472 (17.7) | 1.00 |  |
|  | Renal disease | 33 (24.8) | 1.53 (1.02 – 2.30) | 0.038 |
|  | No dementia^†^ | 482 (17.8) | 1.00 |  |
|  | Dementia | 23 (27.7) | 1.77 (1.09 – 2.90) | 0.020 |
|  | No diabetes mellitus^†^ | 354 (16.2) | 1.00 |  |
|  | Diabetes mellitus | 151 (24.5) | 1.67 (1.35 – 2.08) | < 0.001 |
|  | No OSA^‡^ | 349 (20.2) | 1.00 |  |
|  | OSA | 21 (30.0) | 1.70 (1.00 – 2.86) | 0.046 |
|  | No liver cirrhosis^‡^ | 366 (20.7) | 1.00 |  |
|  | Liver cirrhosis | 4 (14.8) | 0.67 (0.23 – 1.94) | 0.456 |
|  | No risk of falls^‡^ | 339 (20.0) | 1.00 |  |
|  | Risk of falls | 31 (30.1) | 1.72 (1.11 – 2.67) | 0.014 |
| **Antipsychotic (n=930)** | Age <75 years | 589 (36.5) | 1.00 |  |
|  | Age ≥75 years | 341 (28.9) | 0.71 (0.60 – 0.83) | < 0.001 |
|  | Female | 433 (30.8) | 1.00 |  |
|  | Male | 497 (35.8) | 1.26 (1.07 – 1.47) | 0.004 |
|  | Surgery | 488 (47.7) | 1.00 |  |
|  | Medicine | 315 (20.0) | 0.27 (0.23 – 0.33) | < 0.001 |
|  | Mental Health | 48 (85.7) | 6.58 (3.08 – 14.0) | < 0.001 |
|  | Palliative care | 79 (56.4) | 1.42 (1.00 – 2.03) | 0.053 |
|  | No CNS disease^†^ | 693 (30.2) | 1.00 |  |
|  | CNS disease | 237 (47.0) | 2.05 (1.68 – 2.49) | < 0.001 |
|  | No respiratory disease^†^ | 667 (34.9) | 1.00 |  |
|  | Respiratory disease | 263 (29.8) | 0.79 (0.67 – 0.94) | 0.007 |
|  | No renal disease^†^ | 897 (33.7) | 1.00 |  |
|  | Renal disease | 33 (24.8) | 0.65 (0.44 – 0.97) | 0.034 |
|  | No dementia^†^ | 886 (32.7) | 1.00 |  |
|  | Dementia | 44 (53.0) | 2.33 (1.50 – 3.61) | < 0.001 |
|  | No diabetes mellitus^†^ | 741 (34.0) | 1.00 |  |
|  | Diabetes mellitus | 189 (30.7) | 0.86 (0.71 – 1.04) | 0.122 |
|  | No OSA^‡^ | 609 (35.2) | 1.00 |  |
|  | OSA | 13 (18.6) | 0.42 (0.23 – 0.77) | 0.004 |
|  | No liver cirrhosis^‡^ | 617 (34.8) | 1.00 |  |
|  | Liver cirrhosis | 5 (18.5) | 0.43 (0.16 – 1.13) | 0.077 |
|  | No risk of falls^‡^ | 594 (35.0) | 1.00 |  |
|  | Risk of falls | 28 (27.2) | 0.69 (0.44 – 1.08) | 0.104 |
| **Tricyclic antidepressant (n=138)** | Age <75 years | 80 (5.0) | 1.00 |  |
|  | Age ≥75 years | 58 (4.9) | 0.99 (0.70 – 1.40) | 0.956 |
|  | Female | 91 (6.5) | 1.00 |  |
|  | Male | 47 (3.4) | 0.51 (0.35 – 0.73) | < 0.001 |
|  | Surgery | 37 (3.6) | 1.00 |  |
|  | Medicine | 91 (5.8) | 1.63 (1.11 – 2.41) | 0.013 |
|  | Mental Health | 3 (5.4) | 1.51 (0.45 – 5.05) | 0.502 |
|  | Palliative care | 7 (5.0) | 1.40 (0.61 – 3.21) | 0.421 |
|  | No CNS disease^†^ | 114 (5.0) | 1.00 |  |
|  | CNS disease | 24 (4.8) | 0.96 (0.61 – 1.50) | 0.841 |
|  | No respiratory disease^†^ | 81 (4.2) | 1.00 |  |
|  | Respiratory disease | 57 (6.4) | 1.56 (1.01 – 2.21) | 0.012 |
|  | No renal disease^†^ | 129 (4.8) | 1.00 |  |
|  | Renal disease | 9 (6.8) | 1.43 (0.71 – 2.87) | 0.318 |
|  | No dementia^†^ | 138 (5.1) | 1.00 |  |
|  | Dementia | 0 (0) | - | - |
|  | No diabetes mellitus^†^ | 103 (4.7) | 1.00 |  |
|  | Diabetes mellitus | 35 (5.7) | 1.21 (0.82 – 1.80) | 0.334 |
|  | No OSA^‡^ | 90 (5.2) | 1.00 |  |
|  | OSA | 2 (2.9) | 0.54 (0.13 – 2.22) | 0.382 |
|  | No liver cirrhosis^‡^ | 92 (5.2) | 1.00 |  |
|  | Liver cirrhosis | 0 (0) | - | - |
|  | No risk of falls^‡^ | 89 (5.2) | 1.00 |  |
|  | Risk of falls | 3 (2.9) | 0.54 (0.17 – 1.74) | 0.296 |

^†^ Documented comorbidity or inferred from prescribed medication using Rx-Risk Comorbidity Index, n=2795

^‡^ Documented comorbidities only, n=1799. Data not available for all patients.

CNS = Central nervous system; OSA = Obstructive sleep apnoea
